# Optic disc edema and visual loss from paracentral acute middle maculopathy mimicking optic neuritis

**DOI:** 10.5935/0004-2749.2023-0185

**Published:** 2024-09-16

**Authors:** Clarissa R. Pereira, Thais S. A. Benassi, Luiz Guilherme M. Mello, Leonardo P. Cunha, Rony Carlos Preti, Leandro C. Zacharias, Eduardo C. de Souza, Mário L. R. Monteiro

**Affiliations:** 1 Division of Ophthalmology, Laboratory of Investigation in Ophthalmology Faculdade de Medicina, Universidade de São Paulo, São Paulo, SP, Brazil; 2 Department of Specialized Medicine, Centro de Ciências da Saúde, Universidade Federal do Espírito Santo, Vitória, ES, Brazil; 3 Universidade Federal de Juiz de Fora, Juiz de Fora, MG, Brazil

**Keywords:** Optic disc, Papilledema, Optic neuritis, Retinal diseases, Diagnosis, differential, Visual acuity, Diagnostic errors

## Abstract

Optic neuritis is an important cause of unilateral and acute visual loss in young adults, but other differential diagnoses should be considered, especially when the disease has an atypical presentation. This report presents the case of a young woman with reduced visual acuity in her right eye, associated with optic disc edema and a relative afferent pupillary defect, that was initially misdiagnosed as optic neuritis and subsequently found to have paracentral acute middle maculopathy, possibly secondary to subtle impending central retinal vein occlusion. This case emphasizes the need to remember that retinal vascular diseases can occasionally mimic optic neuritis. Detailed anamnesis and ophthalmic examination can avoid unnecessary interventions.

## INTRODUCTION

Optic neuritis (ON) is an important cause of acute unilateral visual loss in young adults, which usually requires extensive neurologic investigation and urgent treatment to prevent permanent visual loss^(1)^. However, the differential diagnoses of other ocular and optic nerve conditions are necessary to prevent inadequate management.

Here, we describe the case of a young woman who presented with episodes of transient visual loss followed by permanent visual impairment and central scotoma in her right eye (OD), which was associated with pain, optic disc edema, and a relative afferent pupillary defect (RAPD).

The initial suspicion of ON was dismissed after carefully evaluating the clinical history, ophthalmologic examination, and optical coherence tomography (OCT) that established the diagnosis of paracentral acute middle maculopathy (PAMM).

## CASE REPORT

A 32-year-old Caucasian woman presented to our service after 3 weeks of subacute visual loss in the OD. She reported the occurrence of painless central visual blurring in this eye thrice daily for three consecutive days that lasted up to 60 min, with spontaneous and complete improvement.

On the fourth day, visual blurring persisted, and she began to experience pain with eye movement. She also described paresthesia on the ipsilateral hemiface, dizziness, tinnitus, and worsening vision during exercises, but denied motor disabilities, nausea, vomiting, or hiccups.

Past medical history revealed migraine, long-standing joint pain in her left knee, which was occasionally treated with oral nonsteroidal anti-inflammatory drugs, and oral contraceptive use. Thus, she went to a clinical emergency department where she underwent computed tomography (CT), and the patient was discharged after receiving pain medication. However, visual blurring persisted, and she consulted an ophthalmologist who observed mild disc edema in her OD and some vascular congestion, and she was referred for a neuro-ophthalmologic evaluation. After 2 weeks, she was examined by a neuro-ophthalmologist who reported a best-corrected visual acuity (BCVA) of 20/40 in the OD and 20/20 in the left eye (OS), right RAPD, and mild optic disc edema associated with peripapillary hemorrhages inferiorly in OD. Therefore, ON was considered, and she was urgently referred to our service.

**Figure 1:**
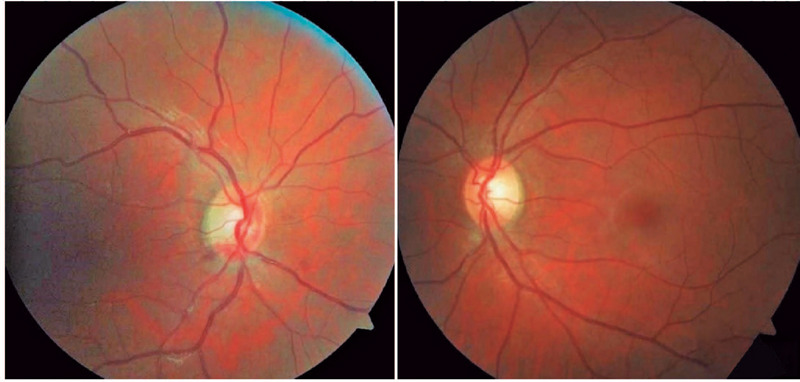
Fundus photograph of the right and left eyes at presentation. Note mild optic disc edema inferiorly, a few peripapillary hemorrhages, and subtle venous congestion in the right eye (left image) compared with the normal left eye (right image).

After 1 week, the patient came to our service reporting some improvement, but she still complained of a central blur. BCVA was 20/20 in both eyes, with difficulty in the OD, and a mild right RAPD was also detected. Eye movements, slit lamp examination, and intraocular pressure were unremarkable. Fundus examination showed mild optic disc edema, few peripapillary hemorrhages, and a discrete pigmentary abnormality in the macula (Figure 1). Compared with the contralateral eye, a small candle-flame-shaped hemorrhage in the superior temporal arcade and mild venous engorgement in the OD were found.

**Figure 2:**
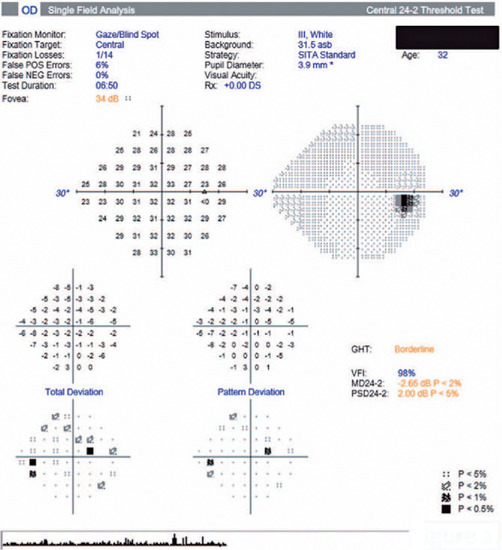
Automated perimetry of the right eye (Humphrey, 24-2 SITA-Standard test) showing scattered points of reduced sensitivity.

Automated visual field analysis showed a small paracentral relative scotoma in the OD (Figure 2) and was unremarkable in the OS. No significant abnormalities were found in fluorescein angiography of both eyes.

**Figure 3:**
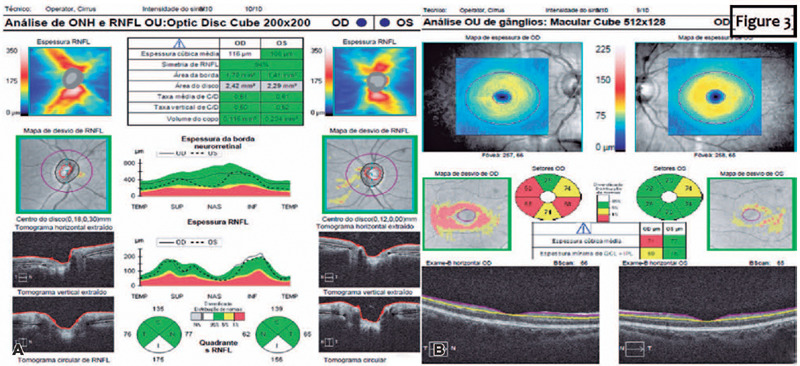
(A) SD-OCT of the peripapillary nerve fiber layer revealing slightly increased thickness in the lower quadrant of the OD, confirming the presence of mild optic disc edema in this eye. (B) SD-OCT of macular ganglion cell layer thickness was diffusely reduced on the right eye and normal on the left eye.

The OCT revealed an increased thickness of the peripapillary retinal nerve fiber layer (pRNFL) and diffuse thinning of the macular ganglion cell layer in OD (Figure 3A), which supported the suspicion of mild optic disc edema (Figure 3B).

**Figure 4:**
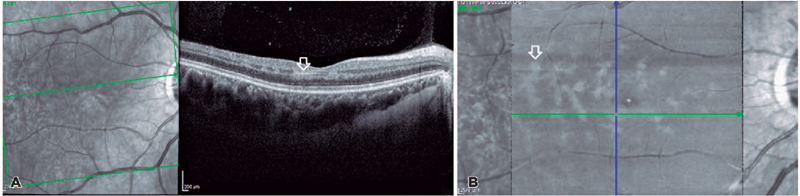
(A) B-scan of the macula exhibiting a hyperreflective band in the INL (white arrow) of the OD, particularly of PAMM. (B) En face OCT imaging shows areas of hyperreflectivity around venules in a fern-like appearance (white arrow), and macular ischemia is observed as irregularity in foveal avascular zone capillaries in OD.

A cross-sectional B-scan of the macula showed a hyperreflective parafoveal band in the inner nuclear layer (INL) of the OD, and the patient was diagnosed with PAMM (Figure 4A). OCT angiography revealed an attenuation of the parafoveal deep vascular complex, reinforcing the hypothesis of PAMM. The en face OCT image showed areas of hyperreflectivity around the venules in a fern-like appearance and an irregularity in the capillaries of the foveal avascular zone, denoting macular ischemia in OD (Figure 4B).

No abnormalities were found in cerebrospinal fluid analysis, brain magnetic resonance imaging with angiography, Doppler ultrasound of the carotid and vertebral arteries, and laboratory results.

## DISCUSSION

Initially, PAMM was described as a variant of acute macular neuroretinopathy (AMN)^(2)^. With the development of spectral-domain OCT (SD-OCT), PAMM was distinguished from AMN because of the preferential involvement of the inner rather than the outer retina, characterized by the development of a parafoveal hyperreflective band in the INL of the retina on OCT^(2^,^3)^.

The mechanism of PAMM is unclear, but middle retinal ischemia may occur due to reduced blood flow in the deep and intermediate capillary plexuses^(2^,^3^,^4^,^5)^. The INL of the retina is a region of a functional watershed zone due to its relative distal location to the inner retinal circulation and choriocapillaris. When the blood supply decreases, the oxygen tension increases in the superficial layers of the retina and choroid, making this intermediate region more susceptible to ischemia^(4)^. In line with this theory, PAMM was frequently associated with other retinal vascular diseases, such as retinal vessel occlusion and sickle cell retinopathy^(6^,^7^,^8)^.

Patients’ complaints are often devalued because of normal visual acuity and subtle fundus abnormalities^(3)^. Therefore, knowing the clinical spectrum of PAMM is important to promote suspicion in the acute phases of the disease, proceeding with an active investigation through SD-OCT, which is essential for the diagnosis^(2^,^3)^.

PAMM has also been associated with other conditions, such as drug intake (amphetamines, vaso-pressors, caffeine, and oral contraceptives), migraine, hypovolemia, orbital compression, viral infections, and vaccination^(2^,^3^,^5)^. Recently, PAMM was associated with SARS-CoV-2 infection and vaccination^(9)^. Notably, COVID-19 increases the risk of vascular thrombotic events, including retinal circulation; thus, PAMM can be a possible complication^(9)^.

Currently, PAMM has no specific treatment, but patients should be educated about controllable risk factors to prevent further vascular events, such as the involvement of the other eye^(10)^.

Although some initial features of our case suggested ON, particularly the presence of optic disc edema and RAPD, other clinical and fundoscopic findings pointed to a vascular etiology. Moreover, OCT was essential to characterize PAMM.

The exact mechanism for the development of the ischemia in our case remains unclear, but it was most likely related to impending central retinal vein occlusion and oral hormonal contraceptive use.

In conclusion, this case documents a possible confusion between ON and retinal vascular disease, emphasizing the importance of a careful evaluation to determine the correct diagnosis of acute visual loss in young patients, avoiding unnecessary testing and treatment.
